# Radical Cure of Rupture

**Published:** 1881-06

**Authors:** 


### RADICAL CURE OF RUPTURE.

The secret method of cure practiced by Dr. George Heaton successfully in one hundred and forty cases is now, after his death, published by Dr. J. H. Davenport. He injected extract of quercus alba into the hernial canal outside the peritoneal sac, to excite a mild degree of irritation in the tendons and fasciæ, so as to lead to contraction. No fatal results followed nor any serious complications. It often cured, and when it failed great relief was obtained, so that a light truss sufficed to support the protrusion.

